# Influencing Factors of Chinese Consumers' Acceptance of Irradiated Food

**DOI:** 10.1002/fsn3.70353

**Published:** 2025-05-28

**Authors:** Gang Li, Zhengkui Zeng, Ke Wang

**Affiliations:** ^1^ CNNC High Energy Equipment (Tianjin) Co. Ltd Tianjin China; ^2^ School of Nuclear Technology and Chemistry & Biology Nuclear Technology Popular Science Education Center Hubei University of Science and Technology Xianning China; ^3^ Wuhan Branch CNNC KaiLi Shenzhen Nuclear Power Service Co. Ltd. Wuhan China

**Keywords:** consumer acceptance, irradiated food, questionnaire survey

## Abstract

Food irradiation enhances food safety and shelf life, but consumer acceptance in China is hindered by radiation‐related misconceptions and low awareness. This study investigated 556 Chinese consumers through an online survey, analyzing demographic influences and attitudes via SPSSAU. The key findings revealed a strong preference for the term “ionized food” (69% acceptance) over “irradiated food” (31%), particularly among those with nuclear risk concerns. This finding highlights the notion that the use of the label “ionized food” can avoid knee‐jerk associations with nuclear radiation. When the term “irradiated food” is employed, clear explanations of what irradiated food involves should be provided on food labels. While over 80% of the respondents recognized conventional sterilization methods (e.g., pasteurization), only 17% identified irradiation. Demographic analysis showed higher initial acceptance among men and the 26–45 age group (75% willingness), although disclosure of everyday irradiated products (e.g., spices, snacks) triggered resistance, especially among women (77% of reversals). Education positively correlated with acceptance, with doctoral students showing 64.5% approval. Notably, 65.6% of initially willing consumers rejected irradiated foods after learning about inconspicuous labeling, reflecting distrust in market transparency. Mobile platforms (WeChat, TikTok) were preferred for science communication (70%), while older adults favored community outreach. Targeted science communication campaigns should be launched to dispel radiation‐related misconceptions. Governments should also customize strategies for different demographic groups, particularly to address the concerns of students, women, and elderly individuals. By leveraging digital media and collaborating with schools, communities, and businesses, consumers can be effectively educated, their concerns can be alleviated, and trust in irradiated food can be enhanced.

## Graphical abstract

Terminology preference: 69% of consumers accept “ionized food” versus 31% for “irradiated food” consumers with negative attitudes toward nuclear reactors. Demographic differences: Women are the most sensitive to irradiated food, 77% shift from being initially willing to buy to being unwilling to buy after being informed the irradiated foods they may daily purchase; the elderly (60+) have the highest rejection rate of irradiated food. Trust challenges: 65.6% of initially willing consumers reject irradiated foods due to unclear labeling, highlighting transparency needs.

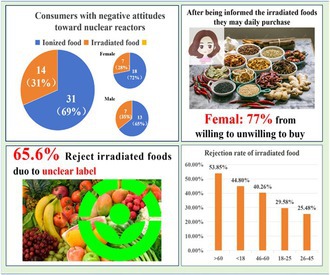

## 1 Introduction

Food irradiation, which is an important food‐processing technology, plays a crucial role in enhancing the safety and hygiene of food while extending its shelf life (Pan et al. 2021). Radioactive materials, including ^60^Co and ^137^Cs, as well as electron and X‐ray beams have been used to irradiate foods (Walter 2012). Although the acceptance of irradiated foods has increased drastically over the past 20 years, many consumers are resistant to them for numerous reasons (Siegrist 2008). This reluctance poses a challenge because individuals' approaches to new technology, lifestyle, preferences, and level of knowledge of irradiated food may be related to their perceptions of food risk (Roosen et al. 2005). Currently, public awareness of food irradiation is low, and many people do not know what irradiation is (Fox et al. 2002). The fear of possible health damage from the consumption of carcinogenic compounds formed during irradiation persists among many consumers worldwide (Withwoth et al. 2017). The majority of this fear stems from the term “irradiation,” which consumers perceive as an addition to food. Additionally, irradiation is associated with the effects of “radiotherapy” or “radiation,” which are closely linked to cancer (Alston et al. 1995).

However, it has been shown that awareness of the nature and benefits of food irradiation leads to positive changes in consumers' perceptions and influences their decision to buy irradiated food. The greater the consumer's awareness of the benefits of irradiation, the greater the probability that irradiated food will be accepted and purchased. Therefore, if information is provided to correct the “knowledge deficit”, consumers will have a more positive attitude toward this technology. Since the successful introduction of the benefits of irradiated foods (such as ground beef), the perception of certain irradiated foods among American consumers has improved (Bruhn 2007; Eustice and Bruhn 2012). However, other studies have contradicted this conclusion (Frewer et al. 2003; Eden et al. 2008). The differences in conclusions are related not only to the degree of consumers' knowledge of irradiated food but also to consumers' culture, gender, occupation, and education level in the region where they are located.

The rapid growth of China's food irradiation industry has positioned it as one of the leading countries in terms of the volume of irradiated food globally (Eustice 2018). The level of acceptance among consumers significantly affects the progression of the irradiated food sector in China. A comprehensive understanding of consumer acceptance can optimize the application of research data to address these concerns effectively. Prior studies have examined consumers' attitudes toward irradiated food, finding that women, elderly individuals, and those with lower educational attainment tend to have less knowledge about irradiated food, harbor more misunderstandings, and exhibit lower acceptance levels, but the rationale behind these attitudes remains unexplored (Wang et al. 2023). This study examines the acceptance of and reasons for Chinese consumers' preference for irradiated food, and it further explores the correlation between acceptance and demographic variables such as gender, age, occupation, and education. To validate the aforementioned conclusions and delve deeper into the genuine factors underlying consumers' attitudes toward irradiated food, this study conducts a more detailed and in‐depth survey among an expanded consumer population. By broadening the sample size and enriching the scope of the investigation, we aim to obtain more comprehensive and robust data. Such data will not only help confirm the previous findings but also uncover nuanced perspectives and underlying mechanisms that influence consumers' perceptions, ultimately providing a more thorough understanding of the dynamics within the food irradiation market and guiding more effective strategies for industry promotion and consumer education.

## 2 Materials and Methods

Before designing the questionnaire for this study, we conducted extensive preliminary science popularization activities. During these activities, we directly interacted with members of the public at various venues, asking them about their attitudes toward irradiated food and the questions that they had. This initial exploration enabled us to gain an in‐depth understanding of consumers' key concerns and psychological perceptions, which served as the foundation for the subsequent questionnaire design.

The questionnaire was created using a WeChat mini‐program, allowing us to take advantage of the platform's wide reach in China. To ensure a diverse and representative sample, we distributed the questionnaire through two of the most widely used social media platforms (WeChat and QQ) in China. Regarding the student group, the majority of participants were undergraduate students at Hubei University of Science and Technology. All consumers were able to complete the questionnaire anonymously online, and throughout the process, we strictly refrained from collecting any personal information to protect their privacy. After data collection was completed, we employed SPSSAU (SPSSPRO 2021), a comprehensive statistical analysis software package, to systematically analyze the collected data, ensuring the scientific rigor and reliability of the research results.

The findings of this study based on the online questionnaire (including questions related to gender, age, occupation, education, and residence.) survey of Chinese consumers nationwide hold significant value. They provide crucial insights for promoting China's food irradiation industry in the consumer market. As shown in Table 1, among the collected responses, 22 were notably shorter (< 30 s), and 15 were notably longer (> 45 min) than the average completion time (2 min), resulting in the exclusion of 37 responses. This exclusion left 556 valid submissions for analysis. Specifically, the results of this study can help foster a more balanced understanding among consumers, enhancing both their knowledge and acceptance of irradiated food. Additionally, the conclusions of this study can serve as a reference for encouraging the broader adoption of food irradiation techniques in the Chinese marketplace, thus contributing to the healthy development of the food irradiation industry in China.

**TABLE 1:** Description of questionnaire questions and options.

Serial number	Question	Options
Q1	Which of the following two food processing names do you prefer? [Single Choice]	Irradiated foods Ionized foods
Q2	What do you know about the common ways of food sterilization? [Multiple Choice]	Moist heat sterilization (e.g., boiling, circulating steam sterilization, autoclaving) Dry heat sterilization (e.g., baking, hot air sterilization) Pasteurization Irradiation sterilization Microwave sterilization Filtration sterilization method Instantaneous high temperature sterilization
Q3	Do you know about irradiated food? [Single Choice]	Excellent understanding Good understanding General understanding No understanding
Q4	How did you come across information about irradiated food? [Single Choice]	School expertise Special science popularization activities in schools Network (WeChat, web page, TikTok) Television or newspapers Outdoor science popularization activities Chat with others
Q5	Would you like to buy irradiated food? [Single Choice]	Very willing Relatively willing Neutral stance Somewhat unwilling Completely unwilling
Q6	Why are you reluctant to buy irradiated food? [Single Choice]	Only pure green and natural food is safe Do not know about irradiated food, dare not buy Irradiated food has nuclear radiation Irradiation will lead to genetic mutations in food Having heard of the dangers of nuclear weapons and nuclear power plant accidents, the word “radiation” reminds people of horrible things The economic cost of irradiated food is high
Q7	The reason you are willing to buy irradiated food? [Single Choice]	I know enough about irradiated food and think there is no harm I believe the safety of irradiated food has been tested by the authoritative department If the taste is good and the price is favorable, it is not important whether the food is irradiated or not Irradiated food has fewer chemical additives and bacteria and a longer shelf life
Q8	If you were informed that food has been irradiated using gamma rays or electrons—rays that are akin to sunlight but with a significantly higher energy level—would you be inclined to purchase irradiated food? [Single Choice]	Very willing Relatively willing Neutral stance Somewhat unwilling Completely unwilling
Q9	Which of the following common foods have you bought or eaten? [Multiple Choice]	Chicken Feet with Pickled Pepper (Junge, Youyou) Boned Duck Feet (Baicaowei, Lulu, Xiaohu Duck) Changsha Chouganzi (Feiwang) Babaoli Qingsi (Feiwang) Sautéed Beef with Chili Sauce Noodles (Master Kong) Spicy Cumin Seasoning Powder (Yilin) None of the above
Q10	If you were informed that the aforementioned foods have undergone sterilization through irradiation, would this information alter your previous purchasing and consumption habits? [Single Choice]	After a clearer understanding, there is no impact It doesn't matter; I would eat as usual I will pay more attention to the packaging information in the future, which will not affect my purchase I haven't bought it before, and I won't buy it Will not buy again due to concerns
Q11	Do you think that widespread dissemination of food irradiation technology is essential? [Single Choice]	Very necessary Relatively necessary Neutral stance Somewhat unnecessary Completely unnecessary
Q12	Which of the following approaches would you most prefer to adopt when engaging in the popularization of irradiated food science? [Single Choice]	Local TV station Radio Mobile media (WeChat, QQ, TikTok) Official newspaper Community science popularization (explanation, small experiment, interesting interaction)
Q13	Please sort the following ways of popularizing science of irradiated food in order. [Sorting Question]	Academic expert College professional teachers College students Government departments Network science popularization big V
Q14	If you were to delve further into the subject of irradiation sterilization, what specific aspects would you like to explore in greater detail? [Single Choice]	Principle and mechanism of irradiation sterilization Application of irradiation sterilization Effect and safety of irradiation sterilization Economic cost of irradiation sterilization Other (please specify)
Q15	Which of these food processing techniques do you find more acceptable? [Multiple choice]	Through the use of food additives Preservation of genetically modified foods High temperature disinfection (by frying, cooking; the flavor of food must be changed) Pickling (salt disinfection, containing a small amount of carcinogen sodium nitrite) Irradiation sterilization (at room temperature, without changing food flavor)

**FIGURE 1:**
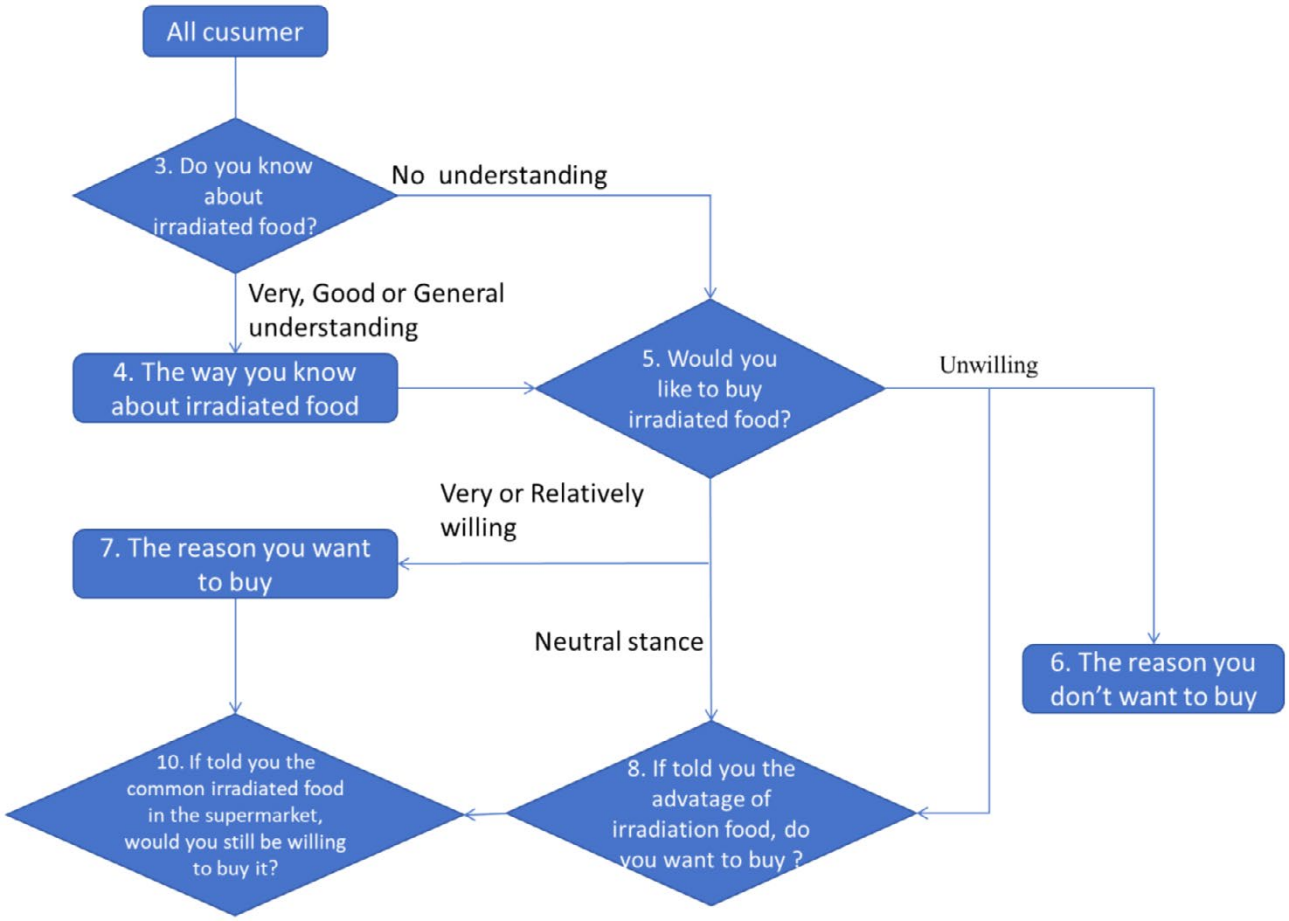
Logic design diagram of the questionnaire.

From a professional perspective, “irradiation” and “ionization” refer to the same scientific concept. However, for ordinary consumers, the term “irradiation” immediately evokes associations with nuclear radiation, carrying inherent risk perceptions. In contrast, “ionization” primarily conjures up imagery of electrical processes, such as the generation of charged particles through electrical interactions, rather than direct risk associations typically linked to radiation. This distinction in semantic interpretation highlights the divergence between technical terminology and layperson perception, where linguistic roots (“radiation” in irradiation vs. “electricity” in ionization) significantly shape initial cognitive associations and emotional responses. Therefore, Question 1 was designed to evaluate consumers' acceptance of the terms “irradiation processing” and “ionization processing”. The subsequent questions aimed to assess the level of consumers' understanding of diverse food processing techniques to shed light on the dissemination of irradiated foods within various consumer demographics. From the third question onward, the survey employed a coherent structure to prevent any contradictions in the respondents' answers, as shown in Figure 1. If the participants selected the “understand” option in Question 3, they were further quizzed in Question 4 about the origins of their knowledge, followed by Question 5, which inquired into their purchasing intentions. Conversely, participants who chose the “do not understand” response in Question 3 bypassed Question 4 and proceeded directly to Question 5, where their purchasing propensity was assessed. This approach facilitated the analysis of the correlation between consumers' comprehension and their desire to purchase. In Question 5, the respondents were segmented into three categories: those who were very or moderately willing to purchase and those who were unwilling (ranging from somewhat to completely unwilling). For those who expressed willingness to purchase, Question 7 examined the rationale behind their preference, followed by Question 9, which informed them about the availability of irradiated foods in everyday life and assessed any subsequent shifts in attitude. For participants who were unwilling to purchase, Question 6 explored the reasons for their reluctance and Question 8 highlighted the benefits of irradiated foods and gauged any alteration in their attitudes. Participants with a moderate willingness to purchase proceeded directly to Question 8, which evaluated any changes in their perceptions. Among the collected responses, 22 were notably shorter, and 15 were notably longer than the average completion time, resulting in the exclusion of 37 responses. This left 556 valid submissions for analysis.

## 3 Results and Analysis

### 3.1 Frequency of Consumer Characteristics

Table 2 illustrates the demographic characteristics of the surveyed population, which comprised 270 males and 286 females. In terms of age, individuals between the ages of 18 and 25 years constituted 48.02% of the sample, those aged 26–45 years represented 28.24%, individuals under 18 years amounted to 7.55%, and individuals over 60 years of age accounted for 2.34%. Students were the predominant group (30.94%), followed by company employees (29.32%), professionals (12.59%), and government officials (9.89%). Approximately 66.19% of the participants had an education level of bachelor's degree or higher, and 75.00% resided in urban areas at the time of the survey.

**TABLE 2:** Frequency of population characteristics.

Name	Option	Frequency	Percentage (%)
Gender	Male	270	48.56
	Female	286	51.44
Age	14–18	42	7.55
	18–25	267	48.02
	26–45	157	28.24
	46–60	77	13.85
	> 60	13	2.34
Occupation	Student	172	30.94
	Professionals (e.g., teacher/doctor/lawyer)	70	12.59
	Service personnel (e.g., catering staff/drivers/salesperson)	29	5.22
	Freelancer (e.g., writer/artist/photographer/tour guide)	30	5.40
	Workers (e.g., factory worker/construction worker/urban sanitation worker)	19	3.42
	Company staff	163	29.32
	Public institution/civil servant/government staff	55	9.89
	Other (businesspeople)	18	3.24
Academic qualifications	Junior college and below	188	33.81
	Undergraduate	314	56.47
	Master's degree	43	7.73
	Doctoral candidate	11	1.98
Total		556	100.0

### 3.2 Validity Analysis

During exploratory factor analysis for validity assessment, the total variance explained (Table 3) plays a crucial role in evaluating the validity of the factor structure. The results of the Kaiser‐Meyer‐Olkin (KMO) test and Bartlett's test of sphericity were employed to determine the suitability of the datasets for factor analysis. The KMO value exceeded 0.6, indicating that the item variables were sufficiently correlated to proceed with factor analysis. Bartlett's test yielded a significance level of *p* < 0.05, confirming the appropriateness of conducting factor analysis. The initial findings revealed that the first factor before rotation contributed the most to the total variance (32.48%), followed by a gradual decrease in the eigenvalues and contribution rates of the subsequent factors. Notably, the sixth factor contributed 6.94%, and the cumulative percentage reached 100%, indicating that all factors collectively accounted for all variations in the data. The rotated factor structure adhered to the fundamental principles of factor analysis and provided a robust foundation for subsequent steps, such as factor naming, variable attribution, and theoretical interpretation. Consequently, the factor structure of this dataset was deemed reasonable, suggesting that it effectively reflects the internal structure and relationships among the original variables. The objective of the rotation step in factor analysis is to increase the clarity and explanatory power of factor interpretations and ensure that each variable correlates strongly with only one factor. As evidenced by the data, the six factors (Factors 1 through 6) elucidated distinct problem dimensions and exhibited significant loading differences. Moreover, the communalities were all one, indicating that all variables were fully explained without any loss of information. This comprehensive explanation underscores the effectiveness of factor analysis in capturing the underlying structure of the data.

**TABLE 3:** Validity analysis of the questionnaire questions.

Name	Factor load factor						Common degree (common factor variance)
	Factor 1	Factor 2	Factor 3	Factor 4	Factor 5	Factor 6	
Q8	−0.11	0.97	−0.09	0.03	0.01	−0.20	1
Q10	0.96	−0.11	0.50	0.05	0.05	0.25	1
Q13	0.27	−0.23	0.10	0.05	−0.01	0.93	1
Q14	0.04	−0.08	0.99	0.04	0.02	0.08	1
Q16	0.05	0.01	0.02	0.10	0.99	−0.01	1
Q17	0.05	0.03	0.04	0.99	0.10	0.05	1
% of variance explained (before rotation)	32.48%	20.43%	15.32%	13.47%	11.36%	6.94%	—
% of total variance explained (after rotation)	16.87%	33.73%	50.46%	67.15%	83.82%	100%	—
KMO value	0.612						—
*p*	0.000***						—

*** Represents the significance level of *p* < 0.001.

** Represents the significance level of *p* < 0.01.

* Represents the significance level of *p* < 0.05.

### 3.3 Cross Chi‐Squared Analysis

Cross chi‐squared analysis is used to determine the proximity of relationships between multiple sets of data. A *p*‐value less than 0.05 in this analysis indicates a significant correlation between variables. Consequently, this study employed cross chi‐squared tests to further investigate the relationships between population characteristics and specific issues.

#### 3.3.1 Cross Chi‐Squared Analysis of Processed Food and Population Characteristics

**FIGURE 2:**
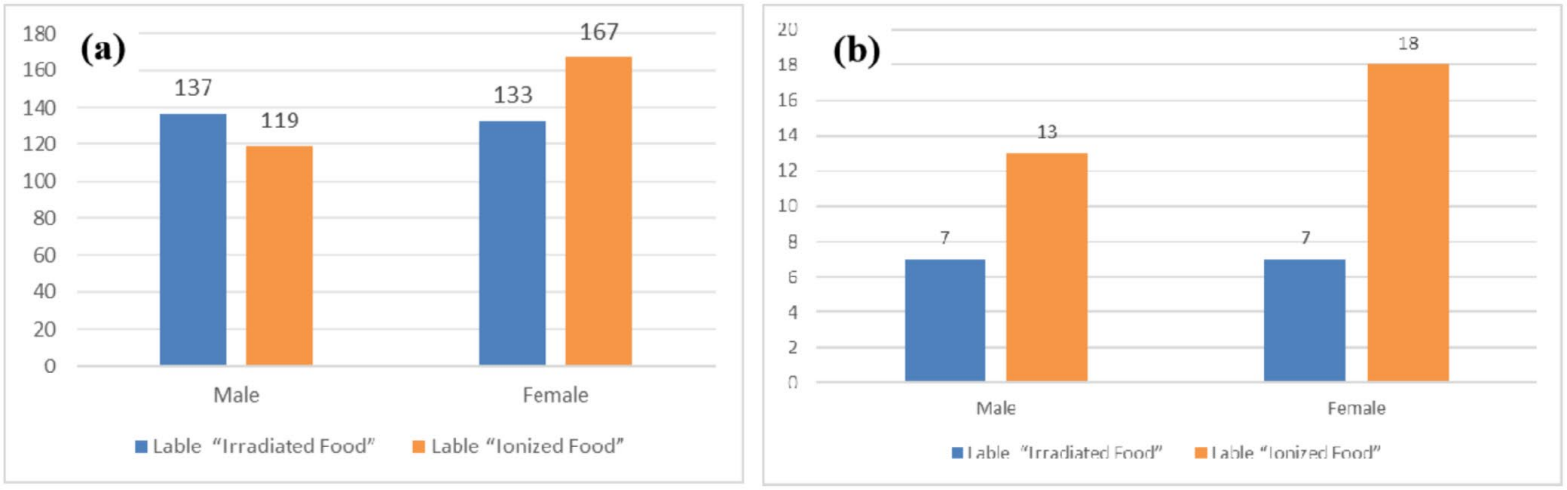
Statistics of food label choices for (a) all consumers and consumers with negative attitudes (b) toward nuclear reactors or the label “radiation”.

Research by Bearth highlighted that the label “irradiated food” negatively impacts consumers' perceptions of the quality of various food products, although “ionized food” exerts a less pronounced effect (Bearth and Siegrist 2019). We conducted an independent survey to further explore this finding. The results revealed that 270 participants preferred the label “irradiated food,” whereas 286 chose “ionized food,” suggesting no significant distinction between the two terms, as shown in Figure 2a. When the data were stratified by consumers with negative attitudes toward nuclear reactors or the label “radiation,” we observed that a greater proportion of consumers favored the term “irradiated processed food” (68.89%) over “irradiated processed food” (31.11%). Consistent with affect heuristics (Finucane et al. 2000; Finucane and Holup 2006), these findings imply that consumers with a more negative attitude toward nuclear power tend to perceive irradiated foods as being of lower quality. Consequently, we focused our analysis on consumers who were unwilling to purchase irradiated food because of their unfavorable views of nuclear reactors or the label “radiation.” As depicted in Figure 2b, 69% of these consumers preferred the label “ionized food.”

#### 3.3.2 Statistics on the Understanding of Sterilization Methods

**FIGURE 3:**
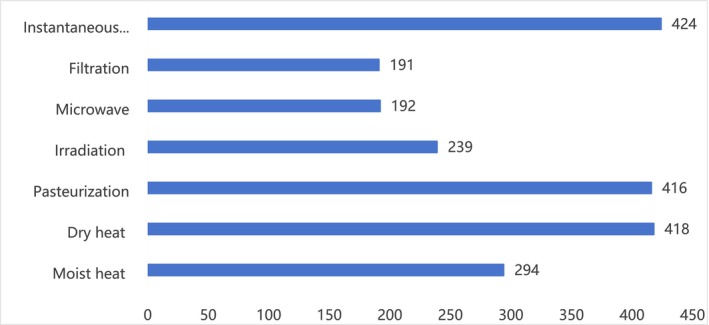
The degree of acceptance of different food processing methods by consumers.

Our survey aimed to evaluate consumers' awareness of several prevalent food‐processing techniques, as presented in Figure 3. The findings revealed that pasteurization was the most widely recognized sterilization method among consumers, followed by moist heat sterilization and dry heat sterilization. In stark contrast, the number of respondents who selected irradiation sterilization was roughly half of the combined total of the first three methods. This finding indicates that there is a relatively low level of awareness regarding irradiation sterilization among the general consumer population.

#### 3.3.3 Cross Chi‐Squared Analysis of Gender and Survey Questions

The results are presented in Table 4. Men exhibited a marginally superior level of knowledge regarding irradiated food compared to women, with percentages of 82.59% and 77.32%, respectively. These values are much higher than those of consumers (10%) in Poland (Buczkowska et al. 2023). Approximately half of the participants of both sexes (46%) expressed their intention to purchase irradiated food. However, among the five common food processing methods provided, only 17.04% of the consumers chose irradiation processing, suggesting hesitation to embrace this method. In terms of public awareness, there was a strong consensus across both genders, with more than 80% of respondents advocating the need to promote irradiated food. Notably, 70% of consumers endorsed mobile platforms such as WeChat, QQ, and TikTok as the preferred medium for scientific dissemination. After being informed that some of the foods that they purchase daily are irradiated foods, there was a notable decrease in apprehension regarding future non‐purchases among men, with a reduction of 21 (25.00%) individuals compared to those unwilling to buy. Conversely, among women, there was a significant increase in concerns about future non‐purchases, with an increase of 22 (19.64%) individuals compared to those who were initially reluctant to purchase. With respect to food‐processing methods, female consumers tended to favor antimicrobial techniques involving food additives, whereas male consumers preferred high‐temperature sterilization. In the early stage of our research, we hypothesized that the low acceptance of irradiated food among women was mainly due to their limited understanding of it. However, the latest research findings indicate that women's heightened sensitivity to irradiated food may be a more plausible underlying factor. Further analysis of the data reveals that among the newly identified group of women reluctant to purchase irradiated food, 98% of respondents cited “irradiated food” as their reason for refusal. This result strongly corroborates the notion that women exhibit a particular sensitivity to the term “irradiation,” leading to a significant shift in their attitudes upon learning that they will be exposed to irradiated food.

**TABLE 4:** Results of cross (chi‐squared) analysis of gender and survey questions.

Topic	Name	Q1 Your gender (%)		Total	*p*
		Male	Female		
Do you know about irradiated food?	Excellent understanding	77 (28.52)	79 (27.62)	156 (28.06)	0.400
	Good understanding	92 (34.07)	97 (33.92)	189 (33.99)	
	General understanding	54 (20.00)	46 (16.08)	100 (17.99)	
	No understanding	47 (17.41)	64 (22.38)	111 (19.96)	
Total		270	286	556	
Would you like to buy irradiated food?	Very willing	65 (24.07)	71 (24.83)	136 (24.46)	0.965
	Relatively willing	62 (22.96)	61 (21.33)	123 (22.12)	
	Neutral stance	59 (21.85)	64 (22.38)	123 (22.12)	
	Somewhat unwilling	64 (23.70)	65 (22.73)	129 (23.20)	
	Completely unwilling	20 (7.41)	25 (8.74)	45 (8.09)	
Total		270	286	556	
If I told you that the above foods were sterilized by irradiation, would you change your previous buying and eating habits?	After a clearer understanding, there is no impact	76 (28.15)	51 (17.83)	127 (22.84)	0.000**
	It doesn't matter; eat as usual	61 (22.59)	60 (20.98)	121 (21.76)	
	I will pay more attention to the packaging information in the future, which will not affect my purchase	70 (25.93)	63 (22.03)	133 (23.92)	
	I haven't bought it before, and I won't buy it	25 (9.26)	25 (8.74)	50 (8.99)	
	Will not buy again due to concerns	38 (14.07)	87 (30.42)	125 (22.48)	
Total		270	286	556	
Do you think it is necessary to popularize food irradiation technology extensively?	Very necessary	104 (38.52)	89 (31.12)	193 (34.71)	0.071
	Relatively necessary	103 (38.15)	142 (49.65)	245 (44.06)	
	Neutral stance	44 (16.30)	39 (13.64)	83 (14.93)	
	Somewhat unnecessary	15 (5.56)	10 (3.50)	25 (4.50)	
	Completely unnecessary	4 (1.48)	6 (2.10)	10 (1.80)	
Total		270	286	556	
Which of the following ways would you prefer to accept for the science popularization of irradiated food?	Local TV station	34 (12.59)	34 (11.89)	68 (12.23)	0.989
	Mobile media (WeChat, QQ, TikTok)	190 (70.37)	205 (71.68)	395 (71.04)	
	Official newspaper	14 (5.19)	14 (4.90)	28 (5.04)	
	Community science popularization (explanation, small experiment, interesting interaction)	32 (11.85)	33 (11.54)	65 (11.69)	
Total		270	286	556	
Which of the following food processing methods is acceptable to you?	Preservative by food additives	56 (20.74)	94 (32.87)	150 (26.98)	0.023*
	Genetically modified foods (genetically modified to improve various resistance)	56 (20.74)	52 (18.18)	108 (19.42)	
	High temperature disinfection (by frying, cooking, etc., the flavor of food must be changed)	96 (35.56)	82 (28.67)	178 (32.01)	
	Pickling (salt disinfection, containing a small amount of carcinogen sodium nitrite)	16 (5.93)	11 (3.85)	27 (4.86)	
	Irradiation sterilization (at room temperature, without changing food flavor)	46 (17.04)	47 (16.43)	93 (16.73)	
Total		270	286	556	

*Note:* * *p* < 0.05 (significant); ** *p* < 0.01 (very significant); *** *p* < 0.001 (most significant).

#### 3.3.4 Cross‐Square Test of Age Versus Survey Question

Table 5 presents the outcomes of the chi‐squared analysis that examined age demographics in relation to survey responses. Notably, a significant majority (93.5%) of consumers aged 26–45 years were cognizant of irradiated food, followed by consumers aged 46–60 (90%). Approximately 60% of the consumers expressed a strong willingness to purchase irradiated food, with the 25–45 age group demonstrating the highest propensity to buy (75%). A total of 54% of elderly individuals explicitly expressed their reluctance to purchase irradiated food. An in‐depth analysis of this group reveals that 57.14% of them held a negative attitude due to a lack of understanding, 28.57% considered irradiated food too expensive, exceeding their consumption expectations, and 5.88% of respondents were concerned about potential genetic risks associated with irradiated food. This result is highly consistent with our previous research hypotheses, further validating the significant impact of cognitive levels on the consumption decisions of the elderly population. When informed that the common foods that they have purchased have been irradiated, 44.8% of consumers under the age of 18 explicitly expressed their unwillingness to purchase irradiated food. A detailed analysis of this group reveals a diverse range of reasons for rejection: 35.29% held a negative attitude due to concerns about potential nuclear radiation risks, genetic mutations, and terrifying things associated with irradiated food; 17.65% considered the products too expensive, exceeding their psychological price expectations; 23.53% liked other food sterilization methods, and another 17.65% refused due to a lack of understanding of irradiated food. In contrast, only 5.88% simply preferred green food, which contributed to their resistance to irradiated food.

**TABLE 5:** Cross (Chi‐squared) analysis results for age and survey questions.

Topic	Name	Q2 Your age (%)					Total	*p*
		Under 18 years old	18–25 years	26–45 years	46–60 years	Over 60 years old		
Do you know about irradiated food?	Excellent understanding	9 (21.43)	63 (23.60)	52 (33.12)	29 (37.66)	3 (23.08)	156 (28.06)	0.000**
	Good understanding	13 (30.95)	71 (26.59)	77 (49.04)	25 (32.47)	3 (23.08)	189 (33.99)	
	General understanding	13 (30.95)	48 (17.98)	18 (11.46)	16 (20.78)	5 (38.46)	100 (17.99)	
	No understanding	7 (16.67)	85 (31.84)	10 (6.37)	7 (9.09)	2 (15.38)	111 (19.96)	
Total		42	267	157	77	13	556	
Would you like to buy irradiated food?	Very willing	6 (14.29)	53 (19.85)	60 (38.22)	15 (19.48)	2 (15.38)	136 (24.46)	0.000**
	Relatively willing	10 (23.81)	55 (20.60)	42 (26.75)	15 (19.48)	1 (7.69)	123 (22.12)	
	Neutral stance	9 (21.43)	80 (29.96)	15 (9.55)	16 (20.78)	3 (23.08)	123 (22.12)	
	Somewhat unwilling	6 (14.29)	67 (25.09)	30 (19.11)	22 (28.57)	4 (30.77)	129 (23.20)	
	Completely unwilling	11 (26.19)	12 (4.49)	10 (6.37)	9 (11.69)	3 (23.08)	45 (8.09)	
Total		42	267	157	77	13	556	
If I told you that the above foods were sterilized by irradiation, would you change your previous buying and eating habits?	After a clearer understanding, there is no impact	6 (14.29)	72 (26.97)	32 (20.38)	13 (16.88)	4 (30.77)	127 (22.84)	0.000**
	It doesn't matter; eat as usual	9 (21.43)	65 (24.34)	27 (17.20)	17 (22.08)	3 (23.08)	121 (21.76)	
	I will pay more attention to the packaging information in the future, which will not affect my purchase	11 (26.19)	71 (26.59)	29 (18.47)	20 (25.97)	2 (15.38)	133 (23.92)	
	I haven't bought it before, and I won't buy it	10 (23.81)	11 (4.12)	12 (7.64)	15 (19.48)	2 (15.38)	50 (8.99)	
	Will not buy again due to concerns	6 (14.29)	48 (17.98)	57 (36.31)	12 (15.58)	2 (15.38)	125 (22.48)	
Total		42	267	157	77	13	556	
Do you think it is necessary to popularize food irradiation technology extensively?	Very necessary	12 (28.57)	88 (32.96)	59 (37.58)	32 (41.56)	2 (15.38)	193 (34.71)	0.000**
	Relatively necessary	8 (19.05)	132 (49.44)	73 (46.50)	29 (37.66)	3 (23.08)	245 (44.06)	
	Neutral stance	13 (30.95)	38 (14.23)	19 (12.10)	10 (12.99)	3 (23.08)	83 (14.93)	
	Somewhat unnecessary	7 (16.67)	6 (2.25)	3 (1.91)	4 (5.19)	5 (38.46)	25 (4.50)	
	Completely unnecessary	2 (4.76)	3 (1.12)	3 (1.91)	2 (2.60)	0 (0.00)	10 (1.80)	
Total		42	267	157	77	13	556	
Which of the following ways would you prefer to accept if you carry out the science popularization of irradiated food?	Local TV station	11 (26.19)	24 (8.99)	12 (7.64)	19 (24.68)	2 (15.38)	68 (12.23)	0.000**
	Mobile media (WeChat, QQ, TikTok)	15 (35.71)	200 (74.91)	131 (83.44)	46 (59.74)	3 (23.08)	395 (71.04)	
	Official newspaper	6 (14.29)	13 (4.87)	5 (3.18)	4 (5.19)	0 (0.00)	28 (5.04)	
	Community science popularization (explanation, small experiment, interesting interaction)	10 (23.81)	30 (11.24)	9 (5.73)	8 (10.39)	8 (61.54)	65 (11.69)	
Total		42	267	157	77	13	556	
Which of the following food processing methods is acceptable to you?	Preservative by food additives	6 (14.29)	57 (21.35)	62 (39.49)	23 (29.87)	2 (15.38)	150 (26.98)	0.000**
	Genetically modified foods	10 (23.81)	48 (17.98)	28 (17.83)	18 (23.38)	4 (30.77)	108 (19.42)	
	High temperature disinfection (by frying, cooking, etc., the flavor of food must be changed)	11 (26.19)	89 (33.33)	53 (33.76)	24 (31.17)	1 (7.69)	178 (32.01)	
	Pickling (salt disinfection, containing a small amount of carcinogen sodium nitrite)	5 (11.90)	10 (3.75)	5 (3.18)	5 (6.49)	2 (15.38)	27 (4.86)	
	Irradiation sterilization (at room temperature, without changing food flavor)	10 (23.81)	63 (23.60)	9 (5.73)	7 (9.09)	4 (30.77)	93 (16.73)	
Total		42	267	157	77	13	556	

*Note:* * *p* < 0.05 (significant); ** *p* < 0.01 (very significant); *** *p* < 0.001 (most significant).

Among consumers aged 18–25 years, predominantly students, 29.58% clearly indicated their reluctance to buy irradiated food. Specifically, 33.33% adopted a negative stance, primarily stemming from concerns about potential nuclear radiation hazards, terrifying things, and genetic mutations associated with such products. Meanwhile, 16.05% opted for green food instead. Significantly, 35.80% of respondents rejected irradiated food simply due to a lack of knowledge about it. In contrast, only 2.47% cited high costs as the reason for their refusal.

Among consumers aged 25–45 years, after being informed that some of the foods that they purchase daily are irradiated foods, they experienced a significant shift in attitude, with a 29% increase in unwillingness to purchase. A gender cross‐analysis showed that the majority of these individuals were women (22; 19.64%). The proportion of consumers over 60 years of age who were unwilling to buy decreased from 55% to 31%, whereas the proportion of consumers in other age groups who were unwilling to buy also declined slightly. Overall, the change in purchase intention among consumers was minimal.

A vast majority (95%) of consumers aged 18–60 years considered popular science activities necessary. However, approximately 38% of consumers older than 60 perceived such activities as unnecessary, which may be attributed to their reluctance to embrace new knowledge. In terms of preferred dissemination methods for popular science, consumers under the age of 60 predominantly opted for mobile media, followed by television and community‐based science outreach. By contrast, consumers over 60 were more inclined toward community science popularization events. Consumers younger than 25 and older than 60 years exhibited a lukewarm acceptance of irradiated food. However, their attitudes toward it improved after they were informed of its benefits. Consequently, there is a pressing need to enhance scientific communication in this age group. For older consumers, it is essential not only to promote scientific outreach but also to conduct community science popularization activities tailored to their specific needs. Finally, the preference for food processing methods revealed that consumers under 25 and over 60 years of age preferred irradiation sterilization to other methods.

#### 3.3.5 Cross‐Square of Occupations and Survey Questions

Tables 6 and 7 illustrate the findings of the cross‐analyses (chi‐squared test) between occupation and survey responses. The data revealed that employees of public institutions, professionals, and officials were more aware of irradiated food. In contrast, students, businesspeople, and workers demonstrated less understanding of irradiated food, with students having the lowest level of knowledge of the subject.

**TABLE 6:** Results of cross (Chi‐squared) analysis of occupations and survey question 3.

Topic	Name	Q3 Your career								Total	*p*
		Student	Professionals	Service industry practitioners	Freelancer	Workers	Company staff	Public institutions/civil servants/government staff	Others		
**Q3**. Do you know about irradiated food?	Excellent understanding	20	31	10	9	1	58	23	4	156	0.000**
	Good understanding	23	24	9	9	5	92	21	6	189	
	General understanding	48	12	6	8	8	7	9	2	100	
	No understanding	81	3	4	4	5	6	2	6	111	
Total		172	70	29	30	19	163	55	18	556	

*Note:* * *p* < 0.05 (significant); ** *p* < 0.01 (very significant); *** *p* < 0.001 (most significant).

**TABLE 7:** Results of cross (chi‐squared) analysis of occupations and survey questions 5 and 8.

Topic	Name	Q3 Your career								Total	*p*
		Student	Professionals	Service industry practitioners	Freelancer	Workers	Company staff	Public institutions/civil servants/government staff	Others		
**Q5**. Would you like to buy irradiated food?	Very willing	15	17	4	7	0	76	17	0	136	0.000**
	Relatively willing	30	22	2	5	4	44	12	4	123	
	Neutral stance	70	10	7	5	9	8	7	7	123	
	Somewhat unwilling	42	17	11	5	5	29	14	6	129	
	Completely unwilling	15	4	5	8	1	6	5	1	45	
Total		172	70	29	30	19	163	55	18	556	
**Q8**. If I told you that the above foods were sterilized by irradiation, would you change your previous buying and eating habits?	After a clearer understanding, there is no impact.	48	12	6	10	7	25	10	9	127	0.000**
	It doesn't matter; eat as usual.	49	19	10	6	3	15	18	1	121	
	I would pay more attention, but it would not affect my purchase	51	12	5	6	6	40	11	2	133	
	I haven't bought it before, and I won't buy it	10	11	5	5	3	6	6	4	50	
	Will not buy again due to concerns	14	16	3	3	0	77	10	2	125	
Total		172	70	29	30	19	163	55	18	556	

*Note:* * *p* < 0.05 (significant); ** *p* < 0.01 (very significant); *** *p* < 0.001 (most significant).

In the age‐related chi‐squared analysis, we observed a significant shift in the attitudes of consumers aged 26–45 after they were informed about common irradiated foods. However, the occupational chi‐squared analysis revealed that the number of company employees unwilling to purchase increased from 35 (21.47%) to 83 (50.92%). Additionally, the number of professionals unwilling to purchase increased from 21 (30.00%) to 27 (38.57%), whereas other occupations showed a decline, with students experiencing the most significant decrease of 34 (19.77%) consumers. Notably, company employees and professionals, who typically possess greater knowledge of irradiated foods, demonstrated greater resistance. This trend was contrary to the expected correlation between understanding and acceptance. This anomaly might stem from this demographic's frequent exposure to irradiated foods in their daily lives, which likely prompts them to exercise caution during purchases, mirroring the public's “Not in My Backyard” (NIMBY) response to nuclear energy.

Workers and freelancers favored local television stations for science outreach, whereas corporate staff and professionals leaned toward mobile media (such as WeChat, QQ, and TikTok) for information. Students and business owners were more inclined to engage in community‐based science initiatives.

#### 3.3.6 Cross‐Square of Education and Survey Questions

The results of the chi‐squared analysis of education and the survey questions are shown in Table 8. The proportion of consumers with different education levels who did not accept irradiated food was similar, ranging from 27.27% to 36.17%, and unacceptable to consumers with degrees from junior college and below was the highest (36.17%). Doctoral students were more knowledgeable about irradiated food. The proportion of undergraduates who chose not to know was 26.11%, which was significantly higher than the average of 19.96%. The proportion of undergraduates and postgraduates who chose mobile media (WeChat, QQ, TikTok) was higher than the average of 7%. Doctoral students were more inclined toward community science popularization (explanations, small experiments, and interesting interactions). The proportion of doctoral students who chose irradiation sterilization (at room temperature, without changing the flavor of food) was higher than the average level, suggesting that the higher the education level of consumers, the better their acceptance of irradiated food.

**TABLE 8:** Cross (Chi‐squared) analysis results between education and survey questions.

Topic	Name	Q4 Your Education (%)				Total	*p*
		Junior college and below	Undergraduate	Master's Institute	Doctoral candidate		
Do you know about irradiated food?	Excellent understanding	65 (34.57)	73 (23.25)	14 (32.56)	4 (36.36)	156 (28.06)	0.009**
	Good understanding	68 (36.17)	103 (32.80)	16 (37.21)	2 (18.18)	189 (33.99)	
	General understanding	34 (18.09)	56 (17.83)	7 (16.28)	3 (27.27)	100 (17.99)	
	No understanding	21 (11.17)	82 (26.11)	6 (13.95)	2 (18.18)	111 (19.96)	
Total		188	314	43	11	556	
Would you like to buy irradiated food?	Very willing	42 (22.34)	82 (26.11)	10 (23.26)	2 (18.18)	136 (24.46)	0.084
	Relatively willing	44 (23.40)	63 (20.06)	12 (27.91)	4 (36.36)	123 (22.12)	
	Neutral stance	34 (18.09)	78 (24.84)	9 (20.93)	2 (18.18)	123 (22.12)	
	Somewhat unwilling	43 (22.87)	77 (24.52)	7 (16.28)	2 (18.18)	129 (23.20)	
	Completely unwilling	25 (13.30)	14 (4.46)	5 (11.63)	1 (9.09)	45 (8.09)	
Total		188	314	43	11	556	
Will you change your previous buying and eating habits?	After a clearer understanding, there is no impact	47 (25.00)	72 (22.93)	7 (16.28)	1 (9.09)	127 (22.84)	0.085
	It doesn't matter; eat as usual	31 (16.49)	72 (22.93)	15 (34.88)	3 (27.27)	121 (21.76)	
	I will pay more attention to the packaging information in the future, which will not affect my purchase	44 (23.40)	79 (25.16)	7 (16.28)	3 (27.27)	133 (23.92)	
	I haven't bought it before, and I won't buy it	26 (13.83)	19 (6.05)	3 (6.98)	2 (18.18)	50 (8.99)	
	Will not buy again due to concerns	40 (21.28)	72 (22.93)	11 (25.58)	2 (18.18)	125 (22.48)	
Total		188	314	43	11	556	
Carry out extensive popularization of food irradiation technology?	Very necessary	76 (40.43)	99 (31.53)	16 (37.21)	2 (18.18)	193 (34.71)	0.001**
	Relatively necessary	58 (30.85)	167 (53.18)	16 (37.21)	4 (36.36)	245 (44.06)	
	Neutral stance	36 (19.15)	35 (11.15)	8 (18.60)	4 (36.36)	83 (14.93)	
	Somewhat unnecessary	14 (7.45)	8 (2.55)	2 (4.65)	1 (9.09)	25 (4.50)	
	Completely unnecessary	4 (2.13)	5 (1.59)	1 (2.33)	0 (0.00)	10 (1.80)	
Total		188	314	43	11	556	
Which of the following would you prefer?	Local TV station	38 (20.21)	26 (8.28)	3 (6.98)	1 (9.09)	68 (12.23)	0.000**
	Mobile media (WeChat, QQ, TikTok)	112 (59.57)	247 (78.66)	33 (76.74)	3 (27.27)	395 (71.04)	
	Official newspaper	15 (7.98)	9 (2.87)	2 (4.65)	2 (18.18)	28 (5.04)	
	Community science popularization (explanation, small experiment, interesting interaction)	23 (12.23)	32 (10.19)	5 (11.63)	5 (45.45)	65 (11.69)	
Total		188	314	43	11	556	
Which of the following food processing methods is more acceptable to you?	Preservative by food additives	44 (23.40)	92 (29.30)	13 (30.23)	1 (9.09)	150 (26.98)	0.014*
	Genetically modified foods (genetically modified to improve various resistances)	51 (27.13)	48 (15.29)	8 (18.60)	1 (9.09)	108 (19.42)	
	High temperature disinfection (by frying, cooking, etc., the flavor of food must be changed)	58 (30.85)	100 (31.85)	16 (37.21)	4 (36.36)	178 (32.01)	
	Pickling (salt disinfection, containing a small amount of carcinogen sodium nitrite)	12 (6.38)	11 (3.50)	2 (4.65)	2 (18.18)	27 (4.86)	
	Irradiation sterilization (at room temperature, without changing food flavor)	23 (12.23)	63 (20.06)	4 (9.30)	3 (27.27)	93 (16.73)	
Total		188	314	43	11	556	

*Note:* * *p* < 0.05 (significant); ** *p* < 0.01 (very significant); *** *p* < 0.001 (most significant).

#### 3.3.7 Cross‐Square Analysis of Knowledge About Irradiated Food

To elucidate the correlation between consumers' willingness to purchase irradiated food and their understanding of it, we conducted a cross‐tabulation analysis, the results of which are shown in Table 9. The proportion of consumers who were completely unwilling, somewhat unwilling, generally willing, and willing (somewhat willing + very willing) to consume irradiated food corresponded to their level of understanding: 6.4% for those with no understanding, 31.1% for those with a general understanding, 64.5% for those with a good understanding, and 60.7% for those with an excellent understanding. These findings suggest a positive correlation between the willingness to purchase and the level of understanding of irradiated food. Typically, consumers with limited knowledge of irradiated food tend to opt for a neutral stance, indicating ambiguity in their attitudes toward this type of food. Our analysis further revealed that consumers with lower levels of understanding of irradiated food are more inclined to show ambiguous purchase intentions. Notably, 47% of consumers who lack knowledge about irradiated food demonstrate a vague willingness to purchase, whereas only 7.74% of consumers with excellent understanding exhibit such ambiguous intentions.

**TABLE 9:** Correlations between consumers' willingness to purchase and their understanding of irradiated food.

Topic	Name	Q8—Do you know about irradiated food? (%)				Total
		No understanding	General understanding	Good understanding	Excellent understanding	
Q5 Would you like to buy irradiated food?	Completely unwilling	8 (7.34)	13 (12.62)	10 (5.29)	14 (9.03)	45 (8.09)
	Somewhat unwilling	42 (38.53)	15 (14.56)	37 (19.58)	35 (22.58)	129 (23.08)
	Generally willing	52 (47.71)	43 (41.75)	20 (10.58)	12 (7.74)	127 (22.84)
	Somewhat willing	5 (4.59)	23 (22.33)	51 (26.98)	40 (25.81)	119 (21.40)
	Very willing	2 (1.83)	9 (8.74)	71 (37.57)	54 (34.84)	136 (24.46)
Total		109	103	189	155	556

In the preceding section, it was observed that a portion of consumers refrained from purchasing irradiated food after gaining knowledge of it through everyday scientific dissemination. Further examination revealed that the majority of these consumers were part of a group comprising 82 individuals who initially expressed a willingness to purchase irradiated food, as shown in Tables 10 and 11. This group accounted for 65.6% of the total number of people who rejected purchasing irradiated food (*n* = 125). Notably, women constituted 77% of this group, with 63 females in the 82‐person cohort. This demographic distribution underscores the heightened sensitivity of women to irradiated food, which aligns with the general perception of women's attitudes toward radiation. The marked shift in consumer sentiment further suggests that their understanding of irradiated food is less comprehensive than they might have assumed. Upon realizing that they consume irradiated food, their stance often shifts to one of resistance. We believe that this reaction stems from consumers' discomfort with unknowingly consuming irradiated food, as in Chinese supermarkets, products such as spices (e.g., cinnamon and star anise) and seasonings (e.g., onions and garlic) are mostly sold in bulk, and supermarkets do not specifically indicate whether they have been irradiated, nor are there any clear irradiation labels. Additionally, for other irradiated packaged snacks, the irradiation labels are often inconspicuous and easily overlooked if consumers do not pay close attention. When we informed them that most of these products had undergone irradiation, it created the impression that the manufacturers were concealing the facts. This impression, in turn, led to misconceptions regarding the safety of irradiated food.

**TABLE 10:** Cross (Chi‐squared) analysis results between gender and survey questions 10.

Name	Q1—Your gender (%)		Total
	Female	Male	
I haven't bought it before, and I won't buy it	8	11	19 (7.14)
I will pay more attention to the packaging information in the future, which will not affect my purchase	25	36	61 (22.93)
It doesn't matter; eat as usual	19	25	44 (16.54)
After a clearer understanding, there is no impact	19	41	60 (22.56)
Will not buy again due to concerns	63	19	82 (30.83)

**TABLE 11:** Cross (Chi‐squared) analysis results between occupation and survey questions 10.

Name	Q1—Your occupation								
	Professionals	Public institutions	Company staff	Other	Student	Worker	Service industry personnel	Freelancer	Total
I haven't bought it before, and I won't buy it	4	3	4	0	3	2	0	3	19
I will pay more attention to the packaging information in the future, which will not affect my purchase	6	7	28	1	14	0	3	2	61
It doesn't matter; eat as usual	13	6	11	0	11	0	2	1	44
After a clearer understanding, there is no impact	7	6	15	2	23	2	1	4	60
Will not buy again due to concerns	9	7	62	1	1	0	0	2	82

#### 3.3.8 Popularization of Irradiated Food Science

The examination of consumers' awareness of irradiated food revealed that 28.06% of respondents were very knowledgeable, 33.93% had a good understanding, and 19.64% were unacquainted with the subject. Notably, among those with knowledge of irradiated food, exposure through school‐based science popularization events, interpersonal discussions, and digital platforms (such as WeChat, web pages, and TikTok) was prevalent. The survey assessing consumers' willingness to purchase irradiated food and the rationale behind their choices, as presented in Table 12, indicated that 46.583% of consumers held a favorable view of buying irradiated food, whereas 31.3% (*n* = 174) were unwilling to purchase. These findings suggest a relatively favorable perception of irradiated food among the Chinese population. When analyzing the reasons for their willingness to purchase irradiated food, 31% of consumers cited their comprehensive understanding of the subject and awareness of its safety, whereas 24.3% believed that the safety of irradiated food had been validated by relevant regulatory authorities. These findings underscore the significant impact of positive authoritative dissemination on shaping consumers' attitudes toward irradiated food. Additionally, 27% of consumers prioritized factors such as price and taste. Conversely, reasons for reluctance to buy irradiated food were as follows: 11.5% of consumers believed that only naturally grown green foods evoke a sense of security, echoing the “natural‐is‐better” reliance observed in previous studies ( Montibeller and Winterfeldt 2015; Siegrist et al. 2016). Furthermore, 24.1% of consumers reported a lack of knowledge about irradiated food, leading to hesitation in purchasing, which can be attributed to innate conservative tendencies toward unfamiliar and novel food (Pliner and Hobden 1992) and novel food technologies (Cox and Evans 2008). Another 15.5% of consumers were concerned about the association between irradiated food and radioactivity, a perception that is detrimental to consumers' quality perceptions, as suggested by previous research. Additionally, 10.9% of consumers were worried about potential genetic mutations in food due to irradiation, reflecting the generally negative public attitude toward genetically modified foods in China. Finally, 13.2% of consumers were influenced by the negative connotations associated with nuclear technology, such as nuclear weapons and power plant accidents, highlighting the significant impact of such news on consumers' perceptions. Among these reasons, all but the first can be attributed to a lack of comprehensive knowledge regarding irradiated food. Targeted scientific dissemination could significantly alter consumers' attitudes by addressing misconceptions and fostering a more informed perspective on irradiated food.

**TABLE 12:** Willingness to buy irradiated food and frequency of reasons.

Name	Options	Frequency	Percentage (%)
Q10 Would you like to buy irradiated food?	Very willing	136	24.46
	Relatively willing	123	22.122
	Neutral stance	123	22.122
	Somewhat unwilling	129	23.201
	Completely unwilling	45	8.094
Q1 1 Why are you reluctant to buy irradiated food? (*n* = 174)	Only pure green and natural food is safe.	20	11.494
	Do not know about irradiated food; dare not buy.	42	24.138
	Irradiated food has nuclear radiation.	27	15.517
	Irradiation will lead to genetic mutations in food.	19	10.92
	Having heard of the dangers of nuclear weapons and nuclear power plant accidents, the word “radiation” reminds people of horrible things.	23	13.218
	The economic cost of irradiated food is high.	17	9.77
Q1 2 Why are you willing to buy irradiated food? (*n* = 259)	I know enough about irradiated food to know there is no harm.	80	30.888
	I believe that the safety of irradiated food has been tested by the relevant departments.	63	24.324
	I care more about the taste and price of the food. If the taste is good and the price is favorable, it is not important whether the food is irradiated or not.	69	26.641
	Irradiated food has fewer chemical additives and bacteria and a longer shelf life.	47	18.147
Total		556	100

Table 13 shows that, after the scientific popularization of the short answer, people's acceptance of irradiated food improved to some extent, but the effect of the change was not obvious. It is necessary to unite governments, enterprises, and schools to actively conduct popular science activities. The internet, school columns, and communities can be used to disseminate the basic concepts and scientific principles of irradiated food to increase public understanding of irradiated food.

**TABLE 13:** Frequency and percentage of attitude change before and after.

Initial attitude	Attitude change	Frequency	Total	Percentage (%)
Generally willing	Very	6	123	4.878
	Willing	31		25.203
	Generally willing	86		69.919
Not really	Very	4	129	3.100
	Willing	11		8.527
	Generally willing	19		14.729
	Not really	95		73.644
Not at all	Willing	2	45	4.444
	Generally willing	1		2.222
	Not at all	42		93.334

## 4 Discussion

The cross‐tabulation analysis of demographic characteristics and survey responses reveals several key findings: There is no significant difference in the selection of irradiated food labels among different groups. However, almost all individuals with a negative attitude toward radiation choose products labeled “ionized food”. Women are the most sensitive to irradiated food, and this sensitivity is evident not only in label selection but also in attitudinal changes. In particular, although most female office workers aged 26–45 claim to understand irradiated food, they actually have a rather biased understanding that is far less comprehensive than they think. Among all groups, seniors over 60 years old have the highest rejection rate of irradiated food, reaching 54%. Among them, for 57.14%, the rejection is due to a lack of understanding of irradiated food, and for 28.57%, the rejection is because of price factors, reflecting the limited information access channels and price sensitivity of the elderly population. The nonacceptance rate of consumers under 18 years old is the second highest at 44.8%, mainly due to their natural fear of radiation, such as concerns about nuclear radiation in food and genetic mutations. The reason may be that they often hear news about nuclear accidents and atomic bomb explosions and thus associate these impressions with irradiated food. The nonacceptance rate of consumers aged 18–25 is 29.58%. For 35.80%, nonacceptance is due to a lack of understanding of irradiated food, and women account for a higher proportion (40%). The impact of factors such as nuclear radiation and genetic mutations accounts for 33.33%.

Based on these conclusions, science popularization efforts should focus on middle‐aged women, students, and elderly individuals, but different strategies are required. For middle‐aged women and students, digital media can be fully utilized for promotion; for elderly individuals, community‐based science popularization activities should be strengthened. In terms of content, for middle‐aged women, the focus should be on introducing common irradiated foods in supermarkets; for students, in addition to explaining the principles of irradiated food processing, it is necessary to dispel their stereotypical impressions associated with the term “nuclear” (such as nuclear radiation, genetic mutations, nuclear accidents); and for elderly individuals, information about the cost‐effectiveness and health benefits of irradiated food should be emphasized.

Negative information about a topic often has a more profound impact on consumers than scientifically based information. Therefore, food industry professionals must be well versed in the scientific rationale behind the irradiation process and its safety implications to advocate for the consumption of irradiated foods while countering misinformation propagated by interest groups or the media (Fox et al. 2002). With respect to the acceptance of science popularization methods, college students exhibited the highest level of acceptance, followed by government departments and online science influencers. This finding suggests that students, government entities, research institutions, and online science influencers can be mobilized to conduct popularization campaigns on irradiated foods after professional training to enhance the credibility of scientific information. Concurrently, it is crucial to regularly assess the effectiveness of science popularization efforts, gather public feedback, and adapt strategies as needed to continually improve the public's acceptance of irradiated foods.

## Author Contributions

**Gang Li:** data curation (lead); investigation (lead); methodology (equal). **Zhengkui Zeng:** investigation (equal); methodology (lead); project administration (lead); resources (lead); supervision (lead); writing – review and editing (lead). **Ke Wang:** data curation (equal); investigation (equal).

## Ethics Statement

This study was conducted in compliance with data protection regulations. The use of personal data was approved by the School of Nuclear Technology and Chemistry & Biology, Hubei University of Science and Technology Ethics Committee, ensuring that all data were anonymized and securely stored.

## Conflicts of Interest

The authors declare no conflicts of interest.

## Data Availability

The data that support the findings of this study are available upon request from the corresponding author. The data are not publicly available due to privacy or ethical restrictions.
